# Subthalamic nucleus stimulation-induced local field potential changes in dystonia

**DOI:** 10.1002/mds.29302

**Published:** 2022-12-23

**Authors:** Christoph Wiest, Francesca Morgante, Flavie Torrecillos, Alek Pogosyan, Shenghong He, Fahd Baig, Ilaria Bertaina, Michael G. Hart, Mark J. Edwards, Erlick A. Pereira, Huiling Tan

**Affiliations:** 1 Medical Research Council Brain Network Dynamics Unit, Nuffield Department of Clinical Neurosciences, University of Oxford, Oxford, UK; 2 Neurosciences Research Centre, Molecular and Clinical Sciences Institute, St. George’s, University of London, London, UK; 3 Institute of Psychiatry, Psychology and Neurosciences, King’s College London, London, UK

**Keywords:** Dystonia, Deep brain stimulation, Subthalamic nucleus, Evoked resonant neural activity, Local field potentials, Finely-tuned gamma oscillations

## Abstract

**Background:**

Subthalamic (STN) stimulation is an effective treatment for Parkinson’s disease and induced local field potential (LFP) changes that have been linked with clinical improvement. Subthalamic stimulation has also been used in dystonia although the internal globus pallidus is the standard target where theta power has been suggested as a physiomarker for adaptive stimulation.

**Objective:**

We aimed to explore if enhanced theta power was also present in STN and if stimulation-induced spectral changes that were previously reported for Parkinson’s disease would occur in dystonia.

**Methods:**

We recorded LFPs from seven patients (twelve hemispheres) with isolated craniocervical dystonia whose electrodes were placed such that inferior, middle and superior contacts covered STN, Zona incerta and thalamus.

**Results:**

We did not observe prominent theta power in STN at rest. Subthalamic stimulation induced similar spectral changes in dystonia as in Parkinson’s disease such as broadband power suppression, evoked resonant neural activity, finely-tuned gamma oscillations and a rise of aperiodic exponents in STN-LFPs. Both power suppression and evoked resonant neural activity localise to STN. Accordingly, single pulse subthalamic stimulation elicits evoked neural activities with largest amplitudes in STN, which are relayed to Zona incerta and thalamus with changing characteristics as the distance from STN increases.

**Conclusions:**

Our results show that subthalamic stimulation-induced spectral changes are a non-disease-specific response to high-frequency stimulation, which can serve as placement markers for STN. This broadens the scope of subthalamic stimulation and makes it an option for other disorders with excessive oscillatory peaks in STN.

## 1 Introduction

Dystonia is a movement disorder characterised by patterned torsional movements, abnormal postures and tremor^1^. The prominent role of the basal ganglia-sensorimotor network in the generation of dystonic symptoms^2^ has led to use of deep brain stimulation (DBS) of the internal globus pallidus (GPi) in the treatment of severe and disabling generalised and segmental dystonia^3^. Theta oscillations in GPi have been associated with dystonic symptoms and suggested as a potential signal for adaptive stimulation in dystonia^4,5^. While GPi remains the DBS target of choice, subthalamic nucleus (STN) stimulation has been reported to be effective in case series of people with focal or generalised dystonia^6^ and cervical dystonia (CD)^7–9^. The rationale for using STN-DBS in dystonia comes from evidence that, besides its well-known effectiveness on bradykinesia, rigidity and tremor, this target is very effective against painful OFF-period dystonia in people with Parkinson’s disease (PD)^10^. Nevertheless, it is unclear if enhanced theta is also present in the STN of people with isolated dystonia.

Subthalamic stimulation in PD is associated with several spectral changes in the STN local field potential (LFP) including beta power suppression^11^, evoked resonant neural activity (ERNA)^12,13^ and finely-tuned gamma (FTG) oscillations^14,15^. These spectral fingerprints have been linked with improvement of parkinsonian symptoms with DBS^16–20^. It is unknown if these same spectral features are present during STN stimulation of people with isolated dystonia, and if so how these relate to lead placement and clinical symptoms. Filling this knowledge gap would help understanding the pathophysiology of isolated dystonia, as well as the mechanism of high-frequency STN stimulation.

Here, we had the unique opportunity to record subthalamic LFPs from seven patients with idiopathic isolated CD implanted with octopolar DBS leads spanning from STN to the ventrolateral thalamus. We asked two main questions. First, how are the power spectra recorded from STN in our patients different from those reported from GPi recordings in isolated dystonia and from STN recordings in PD? Second, are the stimulation-induced phenomena observed in PD also seen in people with dystonia and how do these relate to lead placement?

## 2 Methods

### 2.1 Consent, regulatory approval, patient selection and clinical details

This protocol was approved by the Health Research Authority UK and the National Research Ethics Service local Research Ethics Committee (IRAS: 46576). Seven patients with isolated idiopathic dystonia were recruited at St George’s University Hospitals NHS Foundation Trust, London, and received STN-thalamic dual targeted DBS. Written informed consent was obtained in line with the Declaration of the Principles of Helsinki. Five patients were recorded bilaterally, resulting in a total of 12 STNs included in the study. Clinical details and the tested hemispheres are summarised in Table 1.

### 2.2 Surgery and lead localisation assessment

**Figure 1:**
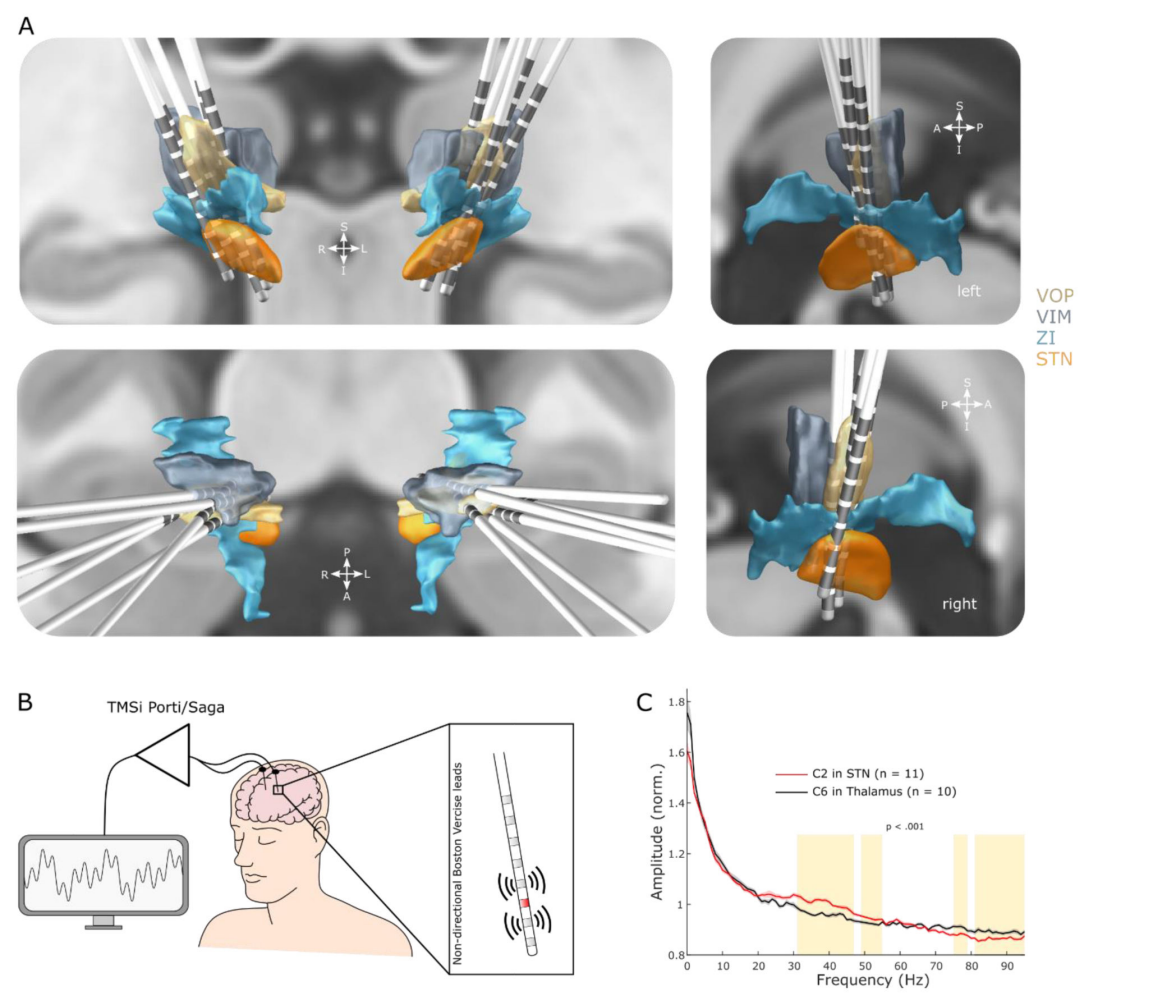
Recording Setup and DBS dual targeting. **A.** Lead reconstructions of all 12 electrodes used in this study. STN: subthalamic nucleus, ZI: Zona incerta, VIM: nucleus ventralis intermedius of the thalamus, VOP: nucleus ventralis oralis posterior of the thalamus. **B.** Leads were temporarily externalised and local field potentials (LFPs) were recorded 4-7 days after implantation using a CE-marked amplifier system. The six middle contact levels were successively stimulated (red), which allowed bipolar LFP recordings from the two adjacent contact levels. **C.** Power spectral densities (PSD, mean ± SEM) from the C2 contact in STN (as judged by Lead-DBS, n = 11 hemispheres) and the C6 contact in ventrolateral thalamus (n = 10) without stimulation. Clusters that are significantly different (cluster-based permutation test, p <.001) between STN and thalamus are highlighted in amber.

The surgical targets were the ventrolateral thalamus (nucleus ventralis intermedius, VIM, and nucleus ventralis oralis posterior, VOP), rostral Zona incerta (rZI) and STN^21^. The non-directional Vercise Standard Lead (model 2201, Boston Scientific Corporation, Marlborough, Massachusetts, USA) with 8 ring contacts (30 cm length, 1.3 mm diameter, 0.5 mm contact spacing, 1.5 mm contact length and 15.5 mm contact span) was implanted such that inferior contacts were placed in STN, superior contacts in ventrolateral thalamus and intervening contacts within or close to the rZI. Electrodes were implanted, externalised and lead trajectories reconstructed (Figure 1A) as described before^13^.

### 2.3 Stimulation and data recording

Recordings were made between 4 and 7 days postoperatively (Table 1), when electrode leads were externalised and patients were OFF all anti-dystonic medication. Monopolar high-frequency stimulation was tested at the 6 middle contacts (C2-C7) as described before^13^. LFPs and EMGs from the affected neck muscles were amplified and sampled at 4096 Hz using a TMSi Saga (TMSi International, Netherlands) and custom-written software developed using the C programming language (Figure 1B).

### 2.4 Experimental paradigm

In patients 1-4 and 6-7 (11 hemispheres), continuous stimulation to each of the 6 middle contacts was delivered at 130 Hz with the intensity increased from 0.5 mA to 4.5 mA or until side effect threshold was reached in steps of 0.5 mA (see Figure 3A). Each DBS block lasted for 46.92 ± 0.99 s (mean ± SEM) separated by resting periods of 27.50 ± 0.59 s. In addition, different stimulation frequencies (100, 130, 150 and 180 Hz for 2 min each) were tested in patient 2 using the contact and current that elicited the most prominent ERNA without side effects. In patients 5-7, single pulse stimulation was subsequently applied at 2 mA (Patient 5) or 4 mA (Patients 6 and 7) to all 8 contact levels (25 pulses to all contacts) and the remaining 7 contacts were recorded in unipolar mode. If not indicated differently, a stimulation frequency of 130 Hz was used.

### 2.5 Signal processing:

#### I Pre-processing and time frequency decomposition

All data analysis was performed using custom-written scripts in MATLAB (version 2020b, The MathWorks Inc., USA). Continuous LFP signals were high-pass filtered at 1 Hz and notch-filtered at 50 Hz (second-order IIR notch filter). Spectral amplitudes were estimated between 1 and 500 Hz using the short-time Fast Fourier transform with a window length of 1 s, 25% overlap of consecutive windows and a Hamming window yielding a frequency resolution of 1 Hz.

#### II Power changes and aperiodic exponents

**Figure 2:**
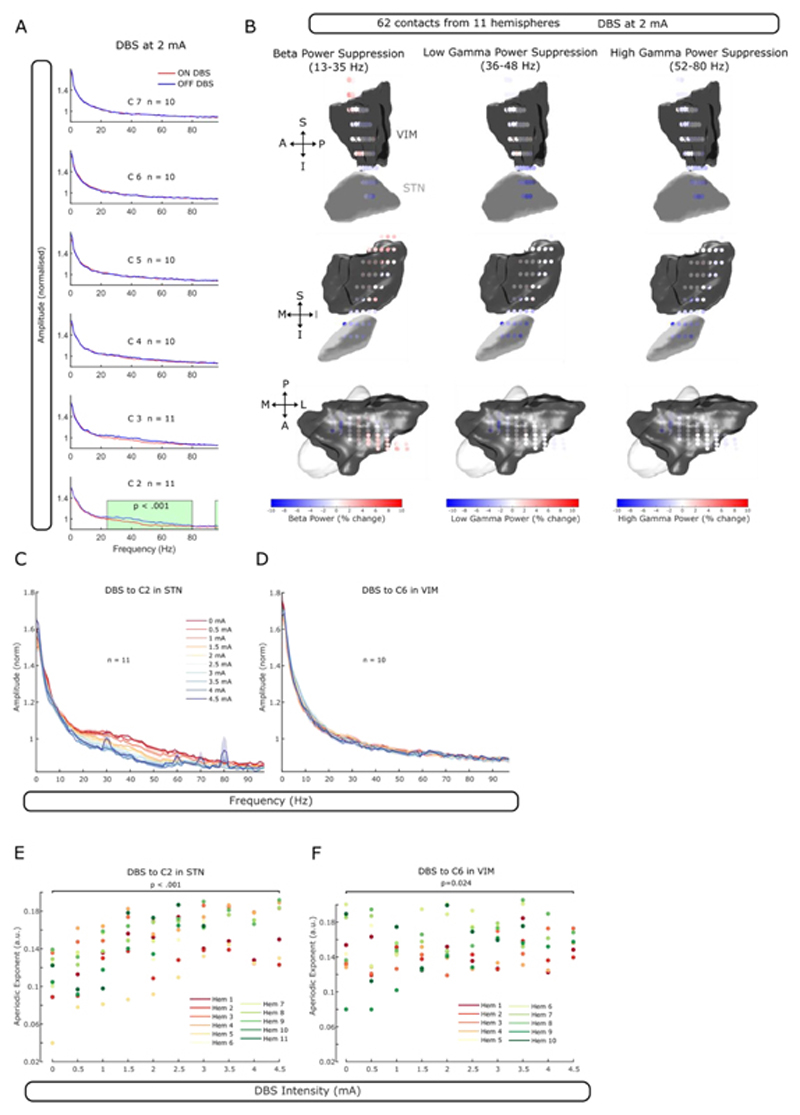
DBS-induced power suppression in STN-LFPs of dystonic patients. **A.** When stimulating contacts C2 to C7 at 2 mA and recording from the adjacent contact pair, average power (mean ± SEM) in the beta (24-35 Hz) and gamma (36-80 Hz) range was suppressed compared to baseline in the most inferior contact only (in STN). Significant clusters are highlighted in green (cluster-based permutation test, p <.001). **B.** Beta (13-35 Hz), low gamma (36-48 Hz) and high gamma (51-80 Hz) power suppression is strongest in the dorsolateral part of STN (n = 62 contacts). **C.** Average power between ~ 20 and ~ 80 Hz is increasingly suppressed in STN with rising DBS intensity. Note the artefacts of stimulation at 4.5 mA manifesting as prominent peaks in different frequency bands (at about 30, 60, 70 and 80 Hz). **D.** No power suppression with increasing DBS intensity when VIM is stimulated. **E.** Aperiodic exponents of the power spectrum increase (1/F slope on a log-log scale becomes steeper) in STN-LFPs with increasing DBS intensity (LME: estimate = 0.006, t = 8.15, p <.001). **F.** Aperiodic exponents of VIM-LFPs only slightly increase with rising DBS intensity (LME: estimate = 0.002, t = 2.30, p =.024). (Hem: hemisphere)

Power spectral densities (PSDs) from 1-95 Hz were calculated for LFPs recorded in bipolar montage and each DBS setting (Figure 2). We defined a 30 s epoch before the first DBS block as baseline. Power around the 50 Hz artefact of mains interference was removed (48-52 Hz) and the gap in the PSD was linearly interpolated. PSDs of both the baseline and all DBS blocks were normalised to the mean power between 1-95 Hz of the baseline PSD. Percentage change in the power relative to the baseline for three frequency ranges were calculated: beta (13-35 Hz), low gamma (36-48 Hz) and high gamma (52-80 Hz).

Aperiodic exponents were isolated from the PSD using the open-source FoooF algorithm (version 1.0.0)^22^. Settings for the algorithm were set as: peak width limits: 2-12; max number of peaks: *infinite*; minimum peak height: 0; peak threshold: 2; and aperiodic mode: *fixed.* Power spectra were parameterised across the frequency range 5 to 50 Hz. The lower bound was selected to avoid the impact of low frequency oscillations, the upper bound was selected to avoid the impact of spectral plateaus at high DBS intensity^23^. The same FoooF settings were used to identify meaningful changes of the aperiodic exponent of STN-LFPs with dopaminergic medication and high-frequency stimulation^24^.

#### III ERNA analysis

**Figure 3:**
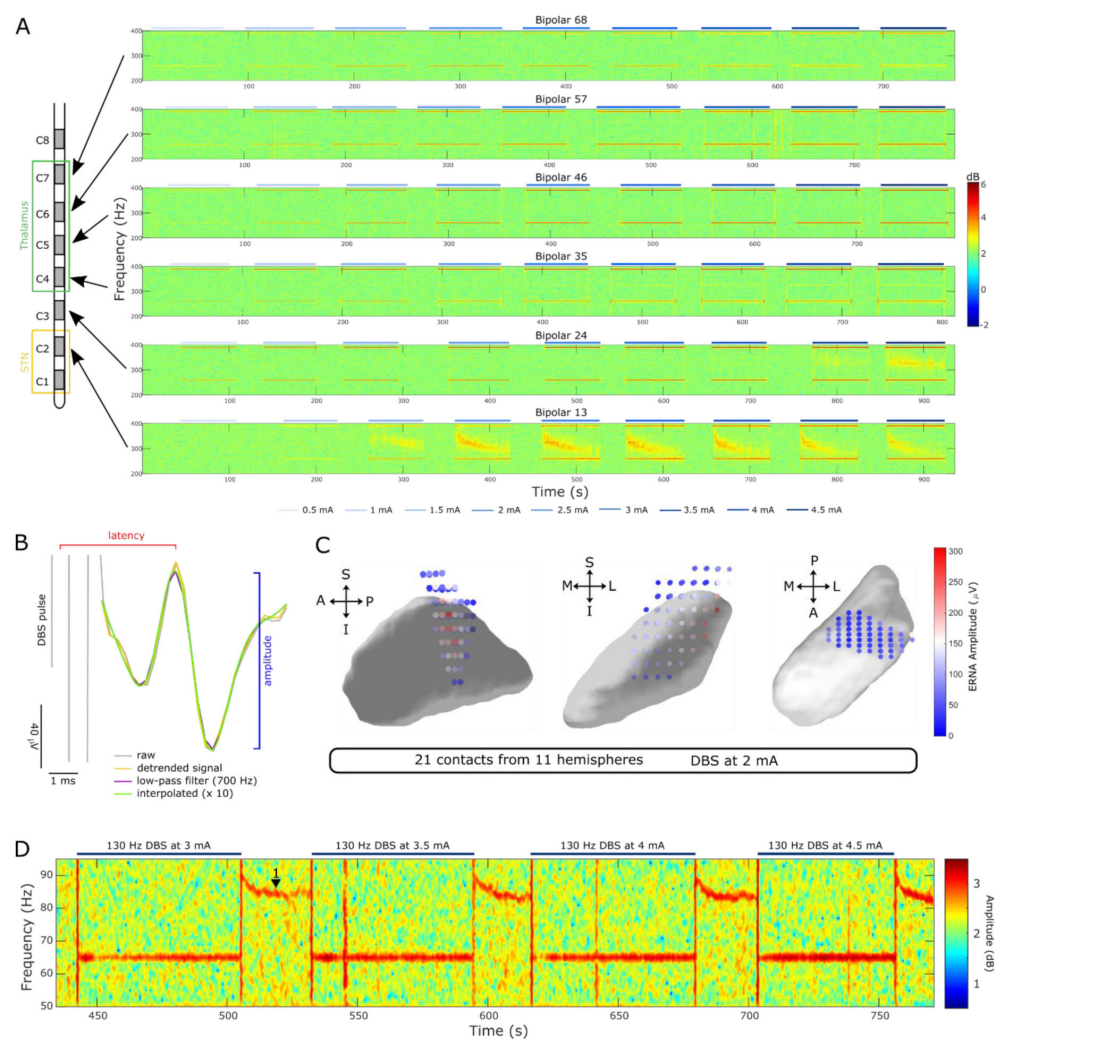
Evoked Resonant Neural Activity (ERNA) and FTG can be elicited by STN-DBS in dystonia. **A.** Stimulating contacts within or close to STN (C2) elicits ERNA recorded from adjacent contacts (C1-C3) with increasing DBS intensity (example spectrograms shown for Patient 1). Note that higher DBS intensity is required to elicit ERNA in contact levels that are further away from a sweet spot in STN (DBS to C3 and recording from C2-C4). Stimulation to any of the more superior contacts (in thalamus) did not elicit ERNA (C4-C7). **B.** Schematic of how ERNA latency and amplitude were calculated based on the time series waveform. The inter-pulse interval was linearly detrended, low-pass filtered at 700 Hz and upsampled using a spline interpolation. **C.** Average ERNA amplitudes of the first 10 pulses at 2 mA stimulation are largest within dorsolateral STN (n = 21; contacts from the right hemisphere were mirrored to the left, individual contact positions were interpolated in 3 D as in Horn et al., 2019) (S: superior, I: inferior, A: anterior, P: posterior, M: medial, L: lateral). The three subplots show the left STN viewed from lateral, anterior, superior, respectively. **D.** Spectrogram with blocks of increasing DBS intensity (3-4.5 mA). DBS-induced FTG (label 1) occurs after the respective DBS blocks (2 of 7 hemispheres) when patients do not exhibit prominent dystonia due to the stun effect.

The presence of the ERNA was assessed in two ways. In the power spectrum, it was defined as a high-amplitude and high-frequency activity which starts at about 350 Hz and decreases in frequency and amplitude with sustained stimulation^13^ (see contact C2, Figure 3A).). In the time series waveform, the ERNA was defined as a high-amplitude evoked response between two stimulation pulses (Figure 3B) which resonates as DBS is switched off or paused^13^.

To map ERNA amplitudes onto subcortical space, stimulation contact positions in MNI space (MNI 152 2009b NLIN ASYM) were extracted from the Lead-DBS pipeline. To calculate ERNA amplitudes, the inter-pulse interval was linearly detrended, low-pass filtered at 700 Hz and upsampled by factor 10 using a spline interpolation (Figure 3B). ERNA amplitudes following the first 10 pulses at 2 mA were averaged since this was the highest intensity that was tested across all hemispheres. Average ERNA amplitudes of the first 10 pulses were assigned to their respective stimulation contacts in MNI space and linearly interpolated in three-dimensional space (Figure 3C). Only contacts with a clear ERNA peak were included in the analysis, which resulted in a total of 21 contacts shown in Figure 3C. Contacts from the right STN were mirrored to the left and are presented in one combined image.

#### IV FTG

The post-DBS FTG was identified after each stimulation block at increasing intensity (see Figure 3D), analysed as described before^25^ and baseline-normalised to the mean amplitude of all power estimates between 1-95 Hz of a 30 s period before the first stimulation block.

#### V Evoked Neural Activity (ENA) after single pulse stimulation

In patients 5-7, we studied evoked neural activities (ENAs) after single pulse stimulation with an average inter-pulse interval of 3.97 ± 0.04 s. To quantify ENAs, LFPs were aligned to individual pulses and averaged. We extracted ENA amplitudes and latencies after every DBS pulse as shown in Figure 3B.

### 2.6 Statistics

Statistical analyses were conducted using custom-written scripts in MATLAB. Linear mixed-effect models were used to assess the effect of stimulation contacts on ENA parameters and aperiodic exponents as described before^13^. To identify clusters of significant power suppression during DBS, we used non-parametric permutation tests with 1000 permutations. Only clusters with p <.001 are highlighted in Figures 1C and 2A.

### 2.11 Data and Code Availability

All data will be available from the Medical Research Council Brain Network Dynamic Unit data sharing platform: https://data.mrc.ox.ac.uk/

## 3 Results

### 3.1 Subthalamic and thalamic power spectral densities in dystonia

We recorded blocks of 37.88 ± 4.86 s at rest without any stimulation from 10 (thalamus) and 11 (STN) hemispheres and did not observe prominent peaks in the theta (4-8 Hz) or beta (13-35 Hz) range in the power spectra of both STN and thalamus (Figure 1C). There was no correlation between muscle activity recorded from the dystonic muscles and LFP power of any frequency range in either STN or thalamus at rest. When comparing power spectra recorded from within or close to STN (recorded from the C2 contact) and thalamus (C6), amplitudes were larger in STN between 31-55 Hz and vice versa from 75-95 Hz (cluster-based permutation test, p <.001, Figure 1C).

### 3.2 DBS induces a broad power suppression in the STN of dystonic patients

Continuous 130 Hz STN-DBS (delivered to the C2 level) at 2 mA induced power suppression over a broad frequency range (25-80 Hz; p <.001; recorded from the adjacent contacts) whereas stimulation to any of the more superior contacts in rZI and thalamus did not lead to significant power changes (in adjacent contacts; Figure 2A). Power suppression of the beta, low gamma and high gamma range was strongest in the dorsolateral part of STN and decreased to all directions (62 contacts from 11 hemispheres included; Figure 2B). In keeping with this, power suppression in STN was more pronounced with increasing DBS intensity (Figure 2C) while increasing DBS currents in VIM/VOP thalamus did not affect the PSD (Figure 2D).

Recently, the aperiodic exponent (1/F slope of the PSD) was suggested as a marker for E/I balance ^24,26,27^. We extracted the aperiodic exponent between 5 and 50 Hz to avoid a spectral plateau at ~ 50 Hz and found that aperiodic exponents in STN increase with rising DBS currents (LME: estimate = 0.006, t = 8.15, p <.001, n = 11 hemispheres), in keeping with the hypothesis that high-frequency stimulation inhibits STN (Figure 2E). This process appears to be non-linear and to flatten out with further increasing currents. In contrast, aperiodic exponents in VIM-LFPs were less affected by increasing intensity of VIM-DBS (LME: estimate = 0.002, t = 2.30, p =.024, n = 10; Figure 2F).

### 3.3 ERNA and finely-tuned gamma can be elicited by STN-DBS in dystonia

When stimulation was delivered at 130 Hz to contacts within or close to the STN (C2), we observed ERNA recorded from neighbouring contacts progressively with increasing DBS intensity in 11 of 12 tested hemispheres (Figure 3A lowest panel). When stimulating the next higher contact (C3), ERNA was still recorded from adjacent contacts, however, only at much higher DBS intensity (e.g. 4.5 mA for Patient 1 left hemisphere). Stimulation to any of the superior contacts in thalamus did not elicit ERNA recorded from surrounding contacts. The observed ERNA shows similar characteristics as reported before in PD: it starts as a high frequency oscillation at ~ 350 Hz and gradually decreases before reaching a steady state after about 1 min^13^.

ERNA has previously been suggested as a placement marker for STN. In support of this, average ERNA amplitudes of the first 10 pulses of 130 Hz DBS (2mA) were largest in dorsolateral STN (Figure 3C) and dropped to all directions with increasing distance from that sweet spot (ERNA from 21 contacts and 11 hemispheres was included).

Levodopa-induced FTG was suggested as a biomarker for dyskinesia in PD^28^. Recently, we reported that FTG can also be induced in STN by DBS alone without dopaminergic medication or dyskinesia in PD patients ^25^. Now, we observed the same phenomenon in two dystonic patients (Figure 3D). Unlike previous reports of FTG in dystonia^29,30^, this FTG activity is *de-novo* DBS-induced and not present at rest. In these patients, DBS-induced FTG appears when stimulation is switched off and decreases in frequency as reported before^25^. We observe DBS-induced FTG in 2 of 7 patients (3 of 12 hemispheres), a similar proportion compared to what was reported in PD^25^. DBS-induced FTG did not correlate with dystonic symptoms or the clinical effect of STN-DBS.

### 3.4 Single pulse stimulation induces ENA and its parameters change with distance from the STN

**Figure 4:**
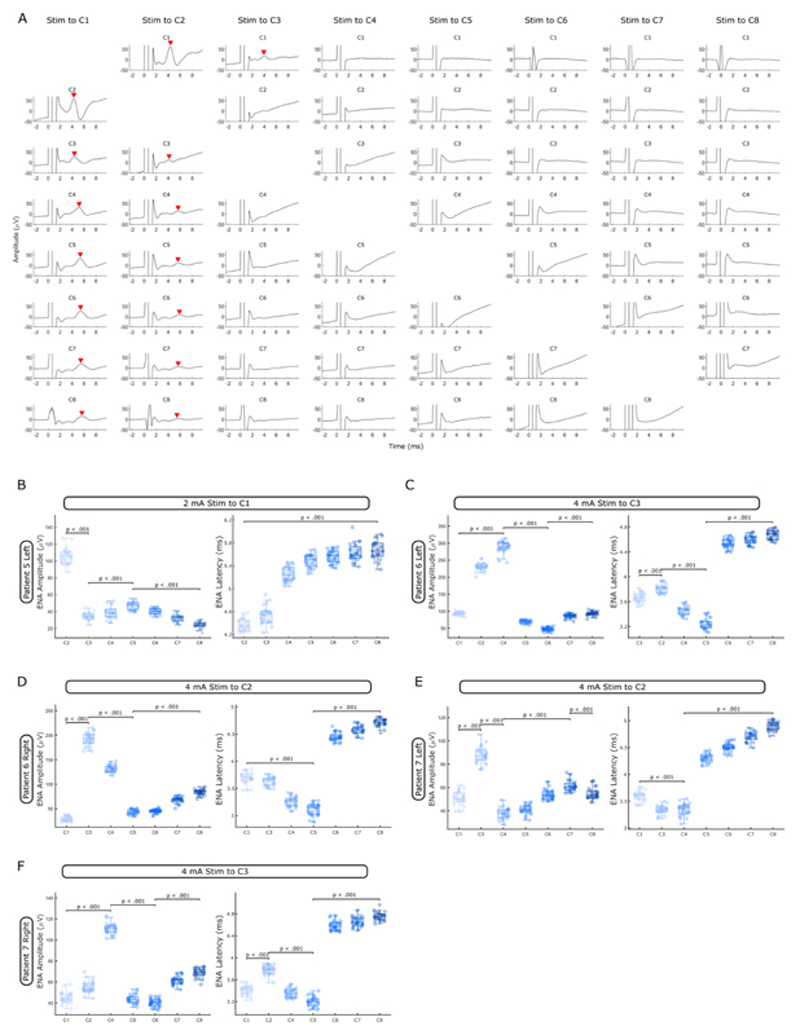
Single pulse STN stimulation elicits Evoked Neural Activity (ENA). **A.** Average ENA responses (mean ± SEM; n = 23 when Stim to C1 and n = 25 for all other columns) after single DBS pulses at 2 mA (ENA peaks are highlighted with red arrowheads, data from Patient 5 left lead). **B.-F.** When stimulating a contact in STN (C1 in B, C2 in D and E, C3 in C and F, as judged by Lead-DBS), ENA amplitudes were largest in the neighbouring contacts (in C-F: superior contact > inferior contact), dropped outside of STN and increased again in thalamus. ENA latencies were shortest in contacts adjacent to stimulation (B) or decreased initially with increasing distance (C-F). Latencies then increased between C3-C4 (B), C5-C6 (C, D and F) and C4-C5 (E), a jump that mirrors the transition from Zona incerta to thalamus. (p-values of linear mixed-effect models are shown)

To test if and to what extent STN-DBS will affect neurons in rZI and thalamus, we applied repetitive single pulse stimulation in Patients 5-7 (Table 1) to all 8 contact levels and recorded LFPs from the remaining 7 contacts in unipolar mode. When aligning and averaging the ENA over successive DBS pulses, we observed a clear ENA peak in all contacts when stimulating the two most inferior contact levels in or close to STN (C1 and C2, Figure 4A). However, the pattern of the ENA changed across different recording contacts. In inferior contacts within or close to STN (C1 + C2), there was an ‘oscillatory’ pattern comprising a peak and a trough. In the remaining contacts (C3-C8), we only observed a peak whose latency increased with distance from STN (LME: estimate = 0.24, t = 24.92, p <.001). This process appeared to be non-linear with the sharpest rise between C3 and C4, which mirrors the transition from rZI to thalamus (Figure 4B). Furthermore, ENA amplitudes changed as a function of distance from STN. When stimulating the most inferior contact in STN (C1), ENA amplitudes (calculated as shown in Figure 3B) were largest in C2, dropped in C3 (LME: estimate = -71.23, t = - 44.72, p <.001) before increasing again up to the C5 contact which is placed in thalamus (LME: estimate = 6.10, t = 9.18, p <.001) (Figure 4B). ENA amplitudes decreased again in any of the more superior contacts (C6-C8) (LME: estimate = -7.63, t = -24.72, p <.001). Changing DBS pulse polarity did not reverse the polarity of the ENA after each pulse. Single pulse stimulation in patients 6 and 7 had similar effects. In these patients, contacts C2 or C3 were placed in STN (as judged by lead reconstructions, see Figure 4C-F). Again, ENA amplitudes were largest in contacts adjacent to stimulation (higher amplitudes in the superior neighbour), then dropped in the middle contacts (C4-C6) and increased again in contacts placed in thalamus (C7-8). ENA latencies were lowest in the middle contact levels (C4-C5) and rapidly increased between C5 and C6 (Figure 4C, D and F) or C4 and C5 (Figure 4E), a jump that mirrors the transition from rZI to thalamus.

## 4 Discussion

Here, we report for the first time stimulation-induced spectral changes of STN activity in people with isolated dystonia. We did not observe prominent theta peaks at rest as has previously been reported in recordings from GPi. However, we found a range of robust stimulation-induced changes in our dystonia cohort that have previously only been reported in PD, such as broadband power suppression, ERNA and DBS-induced FTG, which are specific to STN and not observed in thalamus. ERNA/ENA amplitudes and DBS-induced broadband suppression were largest in STN, supporting their usefulness as markers for lead placement in STN and contact selection in general. Finally, we showed that the aperiodic exponent of STN-LFPs changes with increasing DBS intensity in a way that is reconcilable with the E/I hypothesis. Taken together, these findings point to potential differences in network dynamics in dystonia between GPi and STN, which may be relevant to future work looking at correlations of spectral features with clinical symptoms for adaptive stimulation in dystonia. Furthermore, STN DBS-induced spectral changes reported here may reflect a transdiagnostic pattern of how STN-LFPs respond to high-frequency stimulation. Understanding the origin and temporal dynamics of these changes may open a door to understanding mechanisms underlying high-frequency STN stimulation in general, and therefore its potential application to treatment of other neurological and psychiatric disorders.

### 4.1 Spectral features of the dystonic STN and thalamus

Theta oscillations in GPi^4,5,31–33^ and STN^34^ have been associated with dystonic symptoms and, hence, suggested as a potential signal for adaptive stimulation in dystonia. Here, we did not observe a prominent peak in the theta frequency band of the STN-LFP at rest, which differs from the studies mentioned above in GPi and STN (Figure 1C). Despite the post-operative stun effect as a potential confounder in this study, our results are backed up by a previous report that did not find increased theta power in the STN of dystonia patients, which could be due to differences in input between the GPi and STN^35^. Another recent study indicates that dystonia related spectral changes in the STN may be more prominent during voluntary movements, however, this study investigated dystonia as a motor sign of PD instead of isolated dystonia^36^.

Contrary to the prominent beta peaks in STN that are a hallmark of PD^37,38^, we did not observe a clear beta peak in the STN of dystonic patients (Figure 1C), despite other studies showing beta activity in the STN of dystonic patients at rest^35^ and subthalamic beta peaks in patients with obsessive compulsive disorder^39^. However, we observed larger beta power in STN compared to thalamus. Two previous studies reported higher beta peaks in the GPi-LFP of PD compared to dystonia^33,40^. These disease-specific spectral differences may be explained by the widespread neurodegeneration in PD, which affects basal ganglia neurophysiology and cements excessive beta synchronisation within the basal ganglia as a near pathognomonic marker of bradykinesia and rigidity in PD.

### 4.2 DBS-induced power suppression, ERNA and DBS-induced FTG are not specific to PD

Stimulation-induced changes in STN-LFPs in the beta and gamma range are well studied in PD^13,16^. What is less clear is if these changes are specific to PD or common features of how the STN responds to high-frequency stimulation. Our results support the latter. From the broad (25-80 Hz) subthalamic DBS-induced power suppression (Figure 2A), we can infer that excessive, pathological synchronisation in STN anywhere in this broad spectrum in a particular disease could be flattened by high-frequency STN stimulation. Along with our finding of increasing aperiodic exponents as a function of DBS intensity, we could infer that high-frequency STN-DBS will overall have an inhibitory effect on STN neuronal activity. This might broaden the scope of DBS and make STN-DBS a viable option for other disorders with excessive oscillatory peaks in the STN power spectrum.

As in PD, we also observed ERNA in the STN of dystonic patients. Several attempts have been undertaken to find clinical correlates of the ERNA in PD^13,18,41,42^. While its origin is unclear, it is assumed that the ERNA originates from the effect of STN-DBS on reciprocal STN-GPe (external globus pallidus) connections^18^. Here, we provide evidence that the ERNA is not reliant on PD-specific changes but can also be elicited in the brains of dystonic patients without the widespread neurodegeneration. However, it is still possible, that the ERNA is modulated in the dopamine-deficient brain similar to beta activity.

Levodopa-induced FTG has previously been recorded in PD and linked with the presence of dyskinesia^28^. This link was recently challenged by a study showing that DBS-induced FTG can be observed in PD even without dyskinesia^15,25^. Previously, spontaneous FTG has also been reported in dystonic patients in both cortical and thalamic LFPs, when it was linked with hyperkinetic movements^29,30^. In our cohort, we recorded *de-novo* DBS-induced FTG in STN, which did not vary relative to dystonic postures but appeared reliably after DBS was stopped. This challenges the direct link between DBS-induced FTG with both Parkinsonian and dystonic symptoms and it may rather present a signal of effective STN stimulation^15^, be it epiphenomenal or not.

### 4.3 ERNA/ENA amplitudes and DBS-induced power suppression are largest within STN

Both ERNA^43,44^ and beta power^45,46^ have been suggested as placement markers during surgery and as predictors for the best clinical contact. Our findings of the largest ERNA amplitudes and strongest beta/gamma power suppression in the dorsolateral part of STN (Figure 2B and Figure 3C) are in line with the above studies. Importantly, we show that amplitudes and latencies of ENAs after single DBS pulses are also localised to STN (Figure 4). It is, therefore, not necessary to apply longer bursts of stimulation. Single pulses, which can be applied in a fraction of a second, may be sufficient to optimise lead placement and accelerate contact selection. In general, ENA and ERNA represent the same activity but ERNA has the added resonant effect caused by repetitive stimulation pulses at an inter-pulse interval (frequency) that enhances the amplitude and duration of the ENA (positive interference). Moreover, ENA amplitudes and latencies do not decrease linearly as a function of distance upon stimulation of STN (Figure 4B). ENA amplitudes are largest in STN, second largest in contacts placed in the ventrolateral thalamus and lower in between (see Figure 4B+E). This possibly reflects the thalamic region that receives most inputs from the basal ganglia output structures.

Overall, our results confirm the utility of ERNA/ENA amplitudes and beta/gamma power suppression for both lead placement and contact selection, but challenge the specificity of these spectral changes for PD.

### 4.5 Limitations

Our results may have been confounded by postoperative stun effect, which obscured dystonic symptoms and is known to lower beta activity in PD^47^. Furthermore, we have a relatively low sample size of 7 dystonic patients (12 hemispheres). This is mostly due to the scarcity of externalised DBS patients and externalised dystonic patients with leads in STN are even rarer.

#### Relevant conflicts of interest/financial disclosures

H.T., F.T., A.P., S.H., and C.W. are supported by the Medical Research Council UK (MC_UU_00003/2, MR/V00655X/1, and MR/P012272/1), the National Institute for Health Research (NIHR) Oxford Biomedical Research Centre (BRC), and Rosetrees Trust. S.H. is supported by a Brain Non-clinical Postdoctoral Fellowship. E.A.P. has received speaking honoraria from Boston Scientific and research support from NIHR, UKRI, Life after Paralysis and Rosetrees Trust. F.M. has received speaking honoraria from AbbVie, Medtronic, Boston Scientific, Bial, and Merz; travel grants from the International Parkinson’s Disease and Movement Disorder Society; advisory board fees from AbbVie, Merz, and Boston Scientific; consultancy fees from Boston Scientific, Merz, and Bial; research support from NIHR, UKRI, Boston Scientific, Merz, and Global Kynetic; royalties for the book *Disorders of Movement* from Springer and is a member of the editorial board of *Movement Disorders, Movement Disorders Clinical Practice,* and the *European Journal of Neurology.* M.J.E. has received grant income from NIHR. He has received royalties from the Oxford University Press and honoraria from the International Parkinson’s Disease and Movement Disorder Society and Wiley Publishing. There are no other financial disclosures. The authors declare that there are no conflicts of interest relevant to this work.

## Figures and Tables

**Table 1:** Clinical and recording details. CD: cervical dystonia; FTG: finely-tuned gamma; JRS: Jankovic rating scale; TWSTRS: Toronto Western Spasmodic Torticollis Rating Scale (range 0-35); UL: upper limb; L: left; R: right.

Patient #	Gender (m/f)	Age (yr)	Diagnosis	Disease Duration (yr)	TWSTRS/JRS before DBS	TWSTRS/JRS at recording	Predominant symptoms	Time of Recording (days post-OP)	Data in Figures 2-4 (L/R)	FTG present (L/R)	Single pulse paradigm (L/R)
1	f	64	Segmental dystonia: CD, UL	28	18	4	Tonic posturing of the head	4	L+R	no	no
2	f	49	CD	6	26	16	T onic posturing of the head	4	L+R	L+R	no
3	f	68	Cranio-cervical dystonia	55	3/8	0/1	Blepharospasm	4	R	no	no
4	f	59	CD	5	23	23	Tonic posturing of the head	5	L+R	no	no
5	f	45	Segmental dystonia: CD, UL	25	22	12	Tonic posturing of the head	7	no	L	L
6	m	52	CD	8	24	21	Tonic posturing of the head	7	L+R	no	L+R
7	m	51	Segmental dystonia: CD, trunk	10	26	18	Tonic posturing of the head	7	L+R	no	L+R
